# Influence of rhythmic music game intervention on executive function and sensorimotor ability of children with attention deficit hyperactivity disorder

**DOI:** 10.3389/fpubh.2026.1808386

**Published:** 2026-05-01

**Authors:** Honghui Zhu

**Affiliations:** College of Preschool Education and Arts, Henan Vocational College of Logistics, Zhengzhou, Henan, China

**Keywords:** attention-deficit/hyperactivity disorder, children, clinical symptom assessment, executive function, rhythmic music games, sensorimotor abilities

## Abstract

**Objective:**

To investigate the effectiveness of rhythmic music game–based intervention in improving executive functions and sensorimotor abilities among children with Attention-Deficit/Hyperactivity Disorder (ADHD), thereby providing evidence-based support for non-pharmacological interventions and promoting children’s health.

**Methods:**

A randomized controlled trial was conducted with 64 children diagnosed with ADHD, who were randomly assigned to either an intervention group (*n* = 32) or a control group (*n* = 32). The control group received routine health education, while the intervention group additionally participated in an 8-week rhythmic music game program delivered via tablet devices, consisting of three 40-min sessions per week that included tasks such as beat synchronization and rhythm creation. Executive function and sensorimotor abilities were assessed before and after the intervention using the Stroop Color Word Test (SCWT), Trail Making Test (TMT), and the Test of Gross Motor Development, Third Edition (TGMD-3). At the same time, the attention deficit hyperactivity disorder rating scale (ARS-5) was used to evaluate the severity of the core symptoms of ADHD in children.

**Results:**

In the intervention group, post-intervention assessments showed significant reductions in SCWT word-meaning interference errors (*p* < 0.001) and shorter completion times on TMT Part B (*p* < 0.001). The total score of ARS-5 scale and the scores of each dimension decreased significantly (*p* < 0.001). TGMD-3 total scores also significantly improved (*p* < 0.001), particularly in the locomotor and object-control subdomains reflecting coordination ability (*p* < 0.001). No significant changes were observed in the control group across any measures (*p* > 0.05). Between-group comparisons of pre-post differences revealed statistically significant effects favoring the intervention group (*p* < 0.01).

**Conclusion:**

Rhythmic music game intervention effectively enhances inhibitory control, cognitive flexibility, and sensorimotor coordination in children with ADHD. Given its engaging nature and accessibility, it holds promise as a valuable component within multidisciplinary intervention strategies, offering a practical approach to supporting the healthy development of children with ADHD.

## 1 Introduction

Attention-deficit hyperactivity disorder (ADHD) is one of the most common neurodevelopmental disorders in childhood, and its core symptoms include attention deficit, hyperactivity, and impulsive behavior (1). Epidemiological studies show that the prevalence rate of ADHD among school-age children in the world is about 5–7%, and it is increasing continuously. ADHD not only significantly affects children’s academic performance, social communication, and emotional adjustment ability but also is closely related to high academic failure rates, mental health problems, and the risk of functional impairment in adulthood (2). From the perspective of children’s health and public health, ADHD has become an important issue that needs systematic intervention. How to promote the coordinated development of cognitive and motor functions of ADHD children under the premise of ensuring safety is one of the key challenges in the field of children’s health promotion.

From the perspective of neurological function, ADHD children generally have impaired executive function and abnormal sensory-motor integration (3). The defects of executive function are mainly manifested as insufficient inhibition and control, decreased cognitive flexibility, and limited working memory ability, which directly restrict children’s self-regulation ability of behavior (4). At the same time, neurophysiological studies have found that ADHD children have time delay and abnormal nerve oscillation in the process of sensory information processing and action preparation, especially in alpha and beta bands, which leads to the decline of coordination efficiency between sensory input and motor output (5). This kind of sensory-motor integration disorder not only affects the development of children’s motor skills but also aggravates distraction and impulsive behavior in turn, forming a negative cycle that is not conducive to healthy development. Therefore, it is often difficult to achieve a comprehensive improvement of ADHD children’s core dysfunction by focusing only on behavioral or cognitive intervention (6). At the same time, executive dysfunction, as the core cognitive feature of ADHD, covers the damage of inhibition control, working memory, cognitive flexibility and other dimensions, which is the key factor affecting children’s daily function, and it is difficult to achieve the synergistic improvement of multi-dimensional cognition and motor function by a single intervention method. At present, although there are drug treatments in clinic, 10–30% of children have no response to stimulant drugs or cannot tolerate side effects, and the symptoms are easy to recur after stopping taking drugs. However, the effect of drug therapy on improving executive function and sensorimotor ability of ADHD children is limited, and promoting non-drug intervention and multimodal therapy with the core of improving cognitive and motor function has become a research hotspot.

At present, the clinical intervention of ADHD is still mainly drug therapy, but the long-term application of drug therapy in children is limited by compliance, tolerance, and potential side effects, and some children are prone to rebound after stopping taking drugs (7). Based on this, non-drug interventions, especially multimodal interventions that can simultaneously act on cognitive, sports, and emotional systems, have gradually become the focus of public health and children’s health research. Rhythmic music games, as an intervention form that integrates music rhythm, body movement, and cognitive control training, have strong interest and participation, which conforms to the characteristics of children’s cognitive development (8). Rhythmic stimulation can regulate the time processing mechanism of the nervous system through the auditory-motor pathway, promote sensory-motor integration, and form continuous training for attention maintenance, inhibition control, and cognitive flexibility in game situations, which makes it have unique advantages in ADHD children’s intervention (9).

Although some studies have suggested that music or rhythm-related intervention has a positive effect on ADHD children’s behavior and attention, the existing evidence is still obviously insufficient: on the one hand, the related research focuses on the improvement of symptoms, and the systematic evaluation of key health function indicators such as executive function and sensorimotor ability is relatively limited (10); On the other hand, the intervention forms are mostly unstructured music activities, and there is a lack of standardized gamification schemes based on rhythm characteristics, which limits their popularization in public health (11). Therefore, it is necessary to further verify the influence of rhythmic music game intervention on ADHD children’s core functions under strict research and design and make clear its potential health promotion value.

Stroop Color Word Test (SCWT) and Connection Test (TMT) are classic tools to evaluate the executive function of ADHD children in clinical practice, which are widely used in the diagnosis of cognitive impairment of ADHD and the evaluation of intervention effect. The third edition of the Gross Motor Development Scale for Children (TGMD-3) is the gold standard scale for evaluating children’s sensorimotor ability, which can effectively reflect the improvement degree of abnormal sensorimotor integration in ADHD children. The above scales are commonly used in clinical evaluation of children’s neurodevelopmental disorders, and the evaluation results are highly correlated with the daily function and symptom severity of ADHD children.

Based on this, this study adopts a randomized controlled trial design to systematically investigate the influence of rhythmic music game intervention on executive function and sensorimotor ability of ADHD children. By introducing standardized cognitive tests and motor ability assessment tools, the effectiveness and feasibility of the intervention were evaluated from the perspective of public health and children’s health, aiming at providing a safe, accessible, and potential non-drug intervention path for ADHD children and providing evidence-based comprehensive health intervention strategies for children with neurodevelopmental disorders.

## 2 Methods

### 2.1 Research object

In this study, 64 ADHD children who met the inclusion criteria were selected by convenient sampling method, and the subjects were all children from the pediatric psychological clinic of our hospital. Inclusion criteria: The diagnosis criteria of ADHD in Diagnostic and Statistical Manual of Mental Disorders (Fifth Edition) (DSM-5) were met, and the diagnosis was made jointly by two attending physicians and pediatric psychologists with titles above; Age 6–10 years old, regardless of gender, can understand and cooperate with the game task and evaluation process normally; Parents informed consent and signed the informed consent form, promising to participate in the study and follow-up; In the past 3 months, they have not received targeted non-drug treatment such as music therapy and behavioral intervention, have not taken central stimulants and other drugs that affect nerve function, or have stopped taking drugs for more than 3 months; ARS-5 scale was used to evaluate the core symptoms of ADHD before intervention, and the baseline symptoms were all moderate and severe (the total score of ARS-5 was ≥30). Exclusion criteria: mental retardation, autism spectrum disorder, epilepsy and other neurodevelopmental disorders or mental diseases; There are serious limb dysfunction and hearing impairment, and it is impossible to complete rhythm perception and limb movement tasks; Complicated with serious organic diseases such as heart, liver and kidney; Those who are unable to insist on completing the intervention or evaluation due to force majeure during the study period. In this study, before the sample size was determined, G*Power 3.1 software was used for sample size calculation and statistical power analysis. Taking the number of semantic errors of SCWT as the main outcome index, *α* = 0.05, *β* = 0.2, and the effect amount *d* = 0.8. After calculation, at least 30 samples were needed in each group, and 64 samples were finally determined to be included. The subjects were divided into intervention group and control group by block randomization method, with 32 cases in each group, and the block length was set to 4. Statisticians used computer random number table method to generate random sequences, and implemented distribution hiding. The random distribution results were sealed in opaque envelopes, which were unpacked and distributed by non-researchers when they joined the group. There was no statistical difference between the two groups in gender, age, ADHD subtype (attention deficit type, hyperactivity type, mixed type), and course of disease (*p* > 0.05), which was comparable. This research plan was approved by the hospital ethics committee.

### 2.2 Intervention scheme

The control group received routine health education intervention, and the intervention cycle was the same as that of the intervention group (8 weeks). The specific contents include: conducting an online health lecture once a week (20 min each time), covering daily care skills, diet conditioning, behavior guidance methods, family environment optimization, etc. of ADHD children; Health education manuals are distributed once every 2 weeks, and online consultation services are provided to answer parents’ questions about ADHD children’s care, but no music intervention or targeted cognitive and sports training is provided. The total intervention time is about 80 min, which is a passive health knowledge popularization model.

In the intervention group, on the basis of routine health education in the control group, innovative rhythmic music game intervention was added. The intervention period was 8 weeks, three times a week, 40 min each time, a total of 24 interventions. The total duration of structured and therapist-guided interactive training was about 960 min, and the intervention period was fixed at 9:00–9:40 on weekends. The number of interventions in each group was controlled to be less than 8, and 2 were professionally trained. The core differences between the intervention scheme of this study and the previous cognitive training research based on rhythm or music are as follows: 1. The step-by-step difficulty design is adopted, and the beat complexity and task difficulty are gradually increased in adaptation period, promotion period and consolidation period, which is in line with the development law of children’s cognitive and motor skills; 2. Combining the three-dimensional task mode of “rhythm synchronization+rhythm creation+multi-person cooperation” is not a single rhythm following training, but also realizes the coordination of cognitive training, sports training and social interaction; 3. Develop customized rhythm music game programs based on tablet devices (non-commercial products, independently developed by the research team and computer professionals), accurately combine auditory rhythm stimulation with visual feedback and body movements, and strengthen the activation of “auditory-motor” linkage channels. The main interface of the customized game program is divided into three modules: rhythm task area, visual feedback area and operation area. The rhythm task area displays the rhythm map, rhythm type and creation progress in real time; The visual feedback area presents the synchronization accuracy of action and beat in the form of color and animation (green represents synchronization, red represents non-synchronization, and yellow represents deviation); The operation area is set with three operation modes: touch and click, gesture sliding and body motion induction, which are suitable for different task requirements. The logic flow of the game program is “task loading → rhythm presentation → operation execution → feedback evaluation → difficulty adjustment,” and the subsequent beat complexity and task type are automatically or manually adjusted according to the accuracy of children’s task completion. The intervention adopts flat-panel devices with uniform specifications (screen size 10.9 inches, system version iOS 16.0 and above) and is equipped with customized rhythm music game programs. The game content is designed around two core tasks: beat synchronization and rhythm creation, and the difficulty is gradually increased in stages. The specific intervention contents are as follows:

Adaptation period (week 1–2): Focus on the task of beat synchronization to help children get familiar with game operation and rhythm perception skills. The game scene is set to “rhythm breakthrough,” and the equipment plays music with a fixed beat (the speed is 80–90 beats/min, mainly with clear timbre such as piano and drums). Children click on the corresponding area of the screen according to the music beat or complete physical actions such as clapping and stamping with the beat. The interventionist corrects the accuracy of the action range and beat in real time and warms up (joint activity and simple rhythm) 5 min before each intervention and then 5 min after the intervention.

Promotion period (weeks 3–6): Synchronize the beat and create the basic rhythm, increase the beat speed to 90–100 beats/min, increase the beat changes (such as syncopation and dotted rhythm), and join the multiplayer cooperation mode in the game (two people work together to complete the beat); In the task of rhythm creation, children can choose the timbre of musical instruments through tablet equipment and create a simple rhythm of 2–4 bars based on a given basic beat, and the interventionist gives targeted guidance and encouragement to strengthen the ability of rhythm perception and limb coordination.

Consolidation period (the 7th–8th week): It mainly focuses on complex rhythm creation and comprehensive beat training, and the beat speed is maintained at 100–110 beats/min, incorporating irregular beat elements to increase the interest and challenge of game tasks; The task of rhythm creation is upgraded to 4–8 bars, and children are encouraged to show their own rhythm in combination with body movements. Interveners guide children to evaluate each other and strengthen the intervention effect. Intervention compliance is defined as the course attendance rate, that is, the actual number of interventions/the number of interventions that should be taken ×100%. This study ensures that the intervention compliance is ≥90%. For children who fail to participate in the intervention on time, supplementary interventions will be arranged within 48 h.

### 2.3 Observation index

Before the intervention (baseline, T0) and within 1 week after the intervention (T1), the two groups of children were uniformly evaluated twice. The evaluation was carried out by an appraiser with standardized training, and the appraiser used a blind method to avoid evaluation bias.

Evaluation index of ADHD core symptoms: The fifth edition of the Attention Deficit Hyperactivity Disorder Rating Scale (ARS-5) was used to evaluate the severity of children’s core symptoms, which included two dimensions of attention deficit and hyperactivity impulse, with a total of 18 items. A four-level scoring method was used (0 = completely inconsistent, 3 = completely consistent). The higher the total score, the more serious the symptoms. This scale has good reliability and validity in the evaluation of ADHD children’s symptoms, and is a commonly used clinical ADHD.

Evaluation index of executive function: The Stroop Color Word Test (SCWT) and Connection Test (TMT) are used to evaluate children’s executive function. The specific indicators are as follows: 1. SCWT: The standardized version of the computer is adopted, including three quizzes, namely word meaning reading, color naming, and color word interference. Each quiz contains 60 items, and the number of word meaning errors, color errors, and completion time in the color word interference quizzes are recorded, among which 2. TMT: It is divided into two parts: A and B. TMT- TMT-A requires children to connect scattered numbers in numerical order to evaluate visual search and movement speed; TMT-B requires children to connect in the order of “numbers-letters” to evaluate cognitive flexibility and executive control ability. The core evaluation index is TMT-B completion time (unit: seconds), and the difference between TMT-B and TMT-A completion times is calculated to help reflect cognitive conversion ability. SCWT and TMT are both classic standardized tools for evaluating the executive function of ADHD children in clinical practice, and their evaluation results can directly reflect the degree of cognitive impairment of ADHD children, which is an important reference index for ADHD diagnosis and intervention effect evaluation.

Evaluation index of sensorimotor ability: Children’s sensorimotor ability was evaluated by the third edition of the Test of Gross Motor Development (TGMD-3), which is suitable for children aged 3–10 years, including two dimensions of mobility and manipulation ability, with a total of 13 test items (mobility: running, jumping with one foot, jumping with both feet, etc.; Control ability: throwing the ball, catching the ball, kicking the ball, etc.) Each project is divided into 0–2 points according to the quality of completion (0 points: action cannot be completed; 1 point: partially complete the action; 2 points: skillfully complete the action), and calculate the total score of the scale (out of 26 points) and the scores of each dimension. TGMD-3 is an internationally recognized gold standard scale for evaluating children’s gross motor development. It is widely used to evaluate the sensorimotor ability of children with neurodevelopmental disorders such as ADHD and autism. Its evaluation results are closely related to children’s daily activities and social adaptability, and it is an important tool for clinical evaluation. In the process of evaluation, two appraisers scored independently, and the Kappa value of scoring consistency test was ≥0.85 to ensure the reliability of evaluation results.

### 2.4 Quality control

Strict quality control is implemented throughout the study. Both the interventionist and the appraiser have undergone 2 weeks of standardized training, and they can participate in the study only after passing the examination. The training contents include the operation of the intervention scheme, the use of evaluation tools, and the key points of blind method implementation. Formulate a unified intervention operation manual, define the intervention content, game parameters, and operation specifications at each stage, record and video the whole intervention process, and randomly select 20% of the intervention times for review to ensure the consistency of intervention. All the evaluation data were entered by two researchers. After the entry, the data were compared and corrected, and the database quality verification mechanism was established to eliminate the input errors. Explain the significance of intervention to parents and children before intervention. During the intervention process, children’s participation enthusiasm will be promoted by means of bonus points and game entry incentives. For children who fail to participate in the intervention on time, supplementary intervention will be arranged within 48 h to ensure the compliance of intervention.

### 2.5 Statistical method

Statistical software SPSS 26.0 was used for data processing and analysis. All data were tested for normality (Shapiro–Wilk test) and homogeneity of variance (Levene test). In this study, repeated measurement analysis of variance was used to evaluate the interaction effect between groups (intervention group and control group) and time (before and after intervention), so as to test the real effect of intervention; If the measurement data conforms to the normal distribution, it is expressed by the mean standard deviation (x̄±s), and the paired sample *t-*test is used for comparison at different time points within the group, and the independent sample *t*-test is used for comparison of the difference between the two groups before and after intervention; If it does not conform to the normal distribution, it is represented by median (quartile) [M(Q1, Q3)], and nonparametric test (Wilcoxon signed rank sum test, Mann–Whitney U test) is adopted. According to the medication history, the subjects were divided into two subgroups: non-medication subgroup and drug withdrawal subgroup. The independent sample *t-*test was used to compare the changes of each outcome index before and after the intervention, and the influence of medication history on the intervention effect was analyzed. Counting data were expressed by frequency (rate), and *χ-*test was used for comparison between groups. *p* < 0.05 was statistically significant. At the same time, the missing data were processed by Intentional Treatment Analysis (ITT) to ensure the authenticity and reliability of the research results.

## 3 Results

### 3.1 Comparison of baseline data between the two groups

A total of 64 children with ADHD were enrolled in this study, and were randomly divided into intervention group and control group with 32 cases in each group. According to the medication history, the children were divided into two groups: non-medication subgroup (17 cases in the intervention group and 18 cases in the control group) and drug withdrawal subgroup (15 cases in the intervention group and 14 cases in the control group). There was no statistical difference between the two groups in gender, age, course of disease, subtype, composition ratio of medication history and various evaluation indexes before intervention (*p* > 0.05), which was comparable (Table 1).

**Table 1:** Comparison of baseline characteristics between two groups of children with ADHD (mean ± SD/n, %).

Indicator	Intervention group (*n* = 32)	Control group (*n* = 32)	t/χ^2^ value	*p*-value
Gender (male/female, n)	21/11	20/12	0.103	0.918
Age (years)	9.2 ± 1.5	8.9 ± 1.7	0.782	0.437
Disease duration (years)	2.8 ± 1.1	2.6 ± 1.2	0.654	0.515
ADHD subtype (n, %)			0.256	0.880
Inattentive type	10 (31.25)	11 (34.38)		
Hyperactive–impulsive type	8 (25.00)	7 (21.88)		
Combined type	14 (43.75)	14 (43.75)		
Medication history (n, %)			0.121	0.941
No medication	17 (53.13)	18 (56.25)		
Discontinued medication	15 (46.87)	14 (43.75)		

### 3.2 Effect of rhythmic music game intervention on the results of Stroop Color Word Test (SCWT) for ADHD children

**Figure 1:**
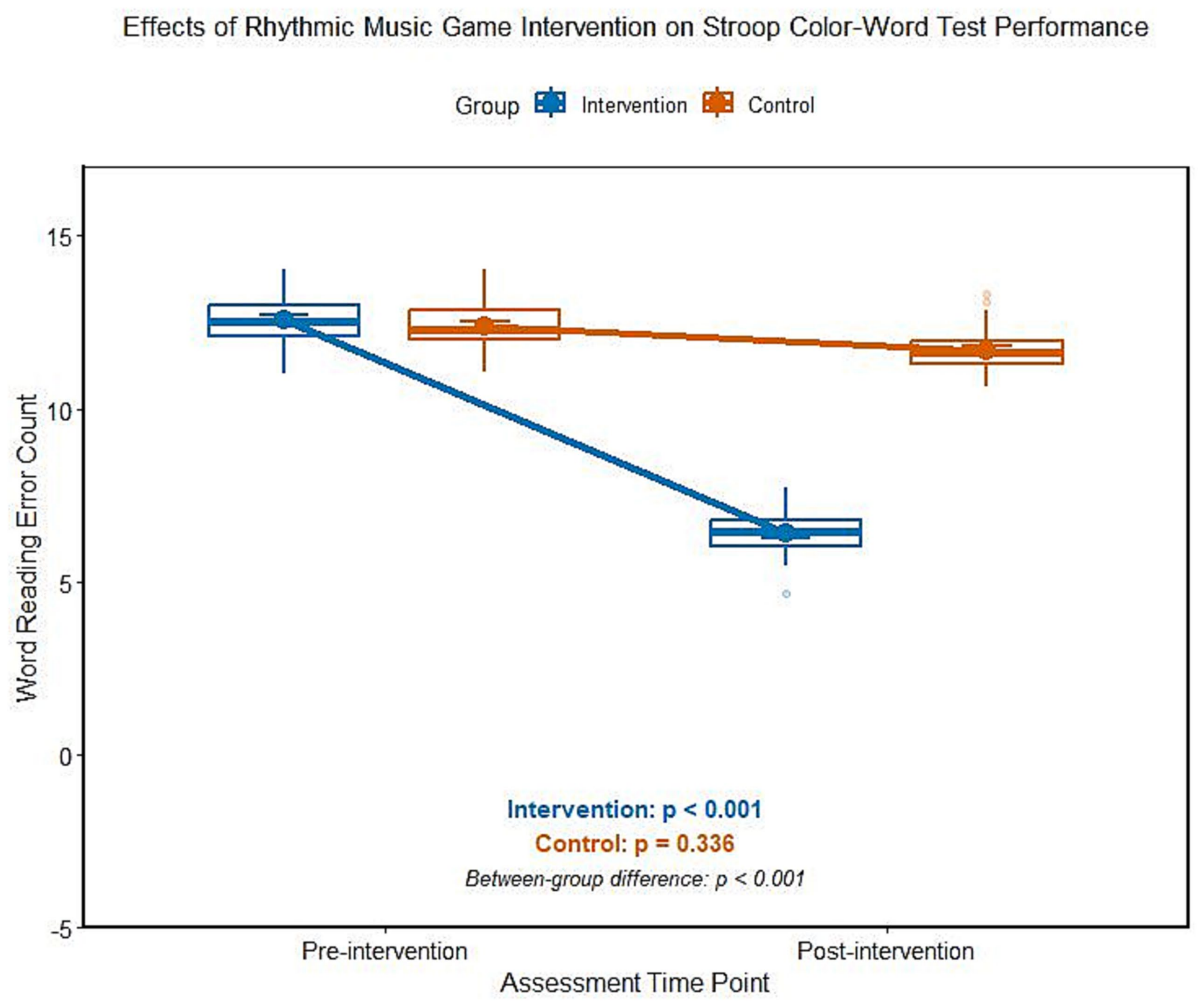
Comparison of the number of semantic errors of SCWT between two groups of ADHD children before and after intervention.

The analysis of variance of repeated measurement shows that there is a significant interaction effect between groups and time on the number of semantic errors of SCWT (*F* = 98.524, *p* < 0.001). After 8 weeks of rhythmic music game intervention, the number of word meaning errors in the intervention group decreased significantly compared with that before intervention, while there was no significant change in the control group before and after intervention, and the difference between the two groups was statistically significant (Table 2; Figure 1). According to the stratified analysis of medication history, there was no significant difference in the reduction of the number of semantic errors of SCWT between the non-medication subgroup and the drug withdrawal subgroup after intervention (t = 0.625, *p* = 0.535).

**Table 2:** Comparison of Stroop Color Word Test (SCWT) results between the two groups (x̄ ± s, items).

Group	Before intervention	After intervention	Difference (after–before)
Intervention group (*n* = 32)	12.6 ± 3.2	6.3 ± 2.1	−6.3 ± 2.5
Control group (*n* = 32)	12.3 ± 3.4	11.8 ± 3.1	−0.5 ± 1.8
Between-group comparison (*t*-value)	0.386	9.257	11.263
Between-group comparison (*p*-value)	0.701	<0.001	<0.001

### 3.3 Influence of rhythmic music game intervention on the results of ADHD children’s connection test (TMT)

**Figure 2:**
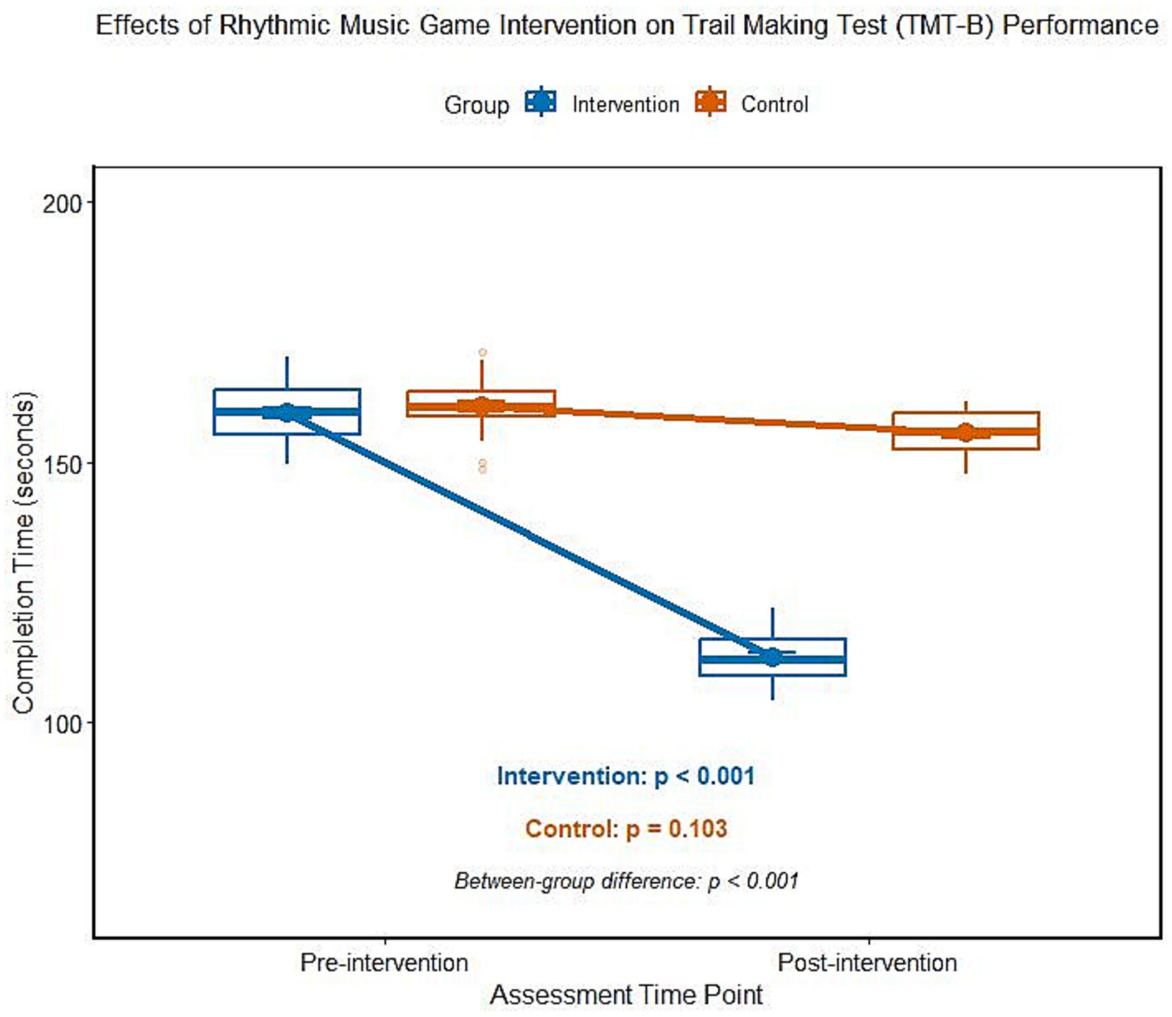
Comparison of TMT-B completion time between two groups of ADHD children before and after intervention.

The analysis of variance of repeated measurements shows that there is a significant interaction between groups and time in the completion time of TMT-B (*F* = 126.845, *p* < 0.001). After the intervention, the completion time of TMT-B in the intervention group was significantly shorter than that before the intervention, and there was no significant difference in the completion time of TMT-B in the control group before and after the intervention, and the difference between the two groups was statistically significant (Table 3; Figure 2). According to the stratified analysis of medication history, there was no significant difference in the shortening of TMT-B completion time between the non-medication subgroup and the drug withdrawal subgroup (*t* = 0.487, *p* = 0.628).

**Table 3:** Comparison of trail making test (TMT) results in children with ADHD between the two groups (x̄ ± s, s).

Group	Before intervention	After intervention	Difference (after–before)
Intervention group (*n* = 32)	158.4 ± 22.6	112.3 ± 18.5	−46.1 ± 16.8
Control group (*n* = 32)	160.2 ± 23.1	155.6 ± 21.8	−4.6 ± 12.3
Between-group comparison (*t*-value)	0.327	8.962	12.057
Between-group comparison (*P-*value)	0.744	< 0.001	< 0.001

The intra-group comparison in the intervention group was *t* = 13.284, *P* < 0.001, the comparison in the control group was *t* = 1.672, *p* = 0.103.

### 3.4 Effect of rhythmic music game intervention on the total score of TGMD-3 of ADHD children

**Figure 3:**
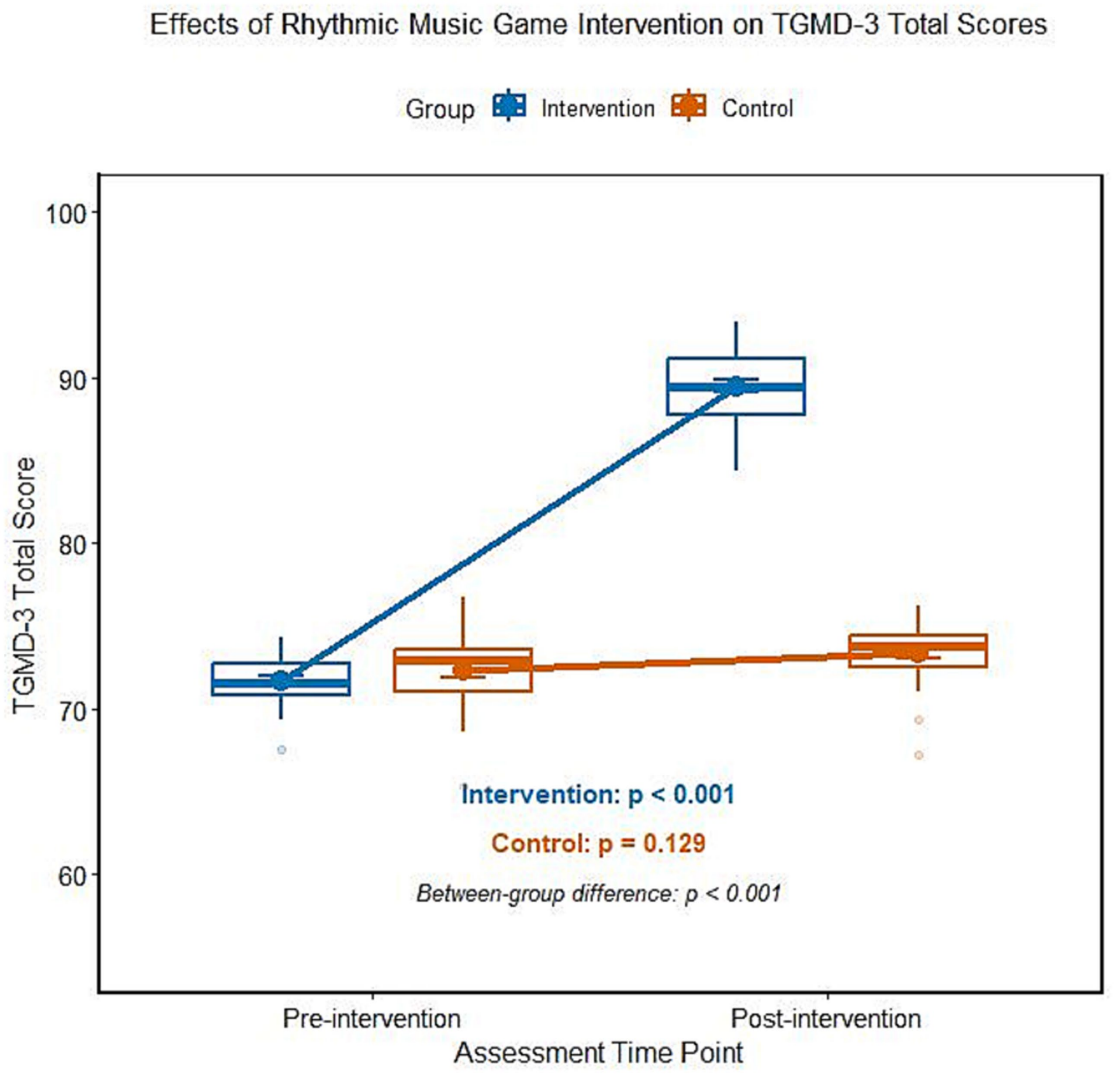
Comparison of TGMD-3 total score between two groups of ADHD children before and after intervention.

The analysis of variance of repeated measurements showed that there was a significant interaction effect between groups and time on the total score of TGMD-3 (*F* = 112.367, *p* < 0.001). The total score of TGMD-3 in the intervention group was significantly higher than that before intervention, while the total score of TGMD-3 in the control group did not change significantly before and after intervention, and the difference between the two groups was statistically significant (Table 4; Figure 3). According to the stratified analysis of medication history, there was no significant difference in the total score of TGMD-3 between the non-medication subgroup and the drug withdrawal subgroup after intervention (*t* = 0.753, *p* = 0.455).

**Table 4:** Comparison of total TGMD-3 scores in children with ADHD between the two groups before and after intervention (x̄ ± s, points).

Group	Before intervention	After intervention	Difference (after–before)
Intervention group (*n* = 32)	72.3 ± 8.5	89.6 ± 9.2	17.3 ± 6.8
Control group (*n* = 32)	71.8 ± 8.9	73.2 ± 9.1	1.4 ± 4.2
Between-group comparison (*t*-value)	0.235	7.834	11.538
Between-group comparison (*p-*value)	0.815	< 0.001	< 0.001

### 3.5 Influence of rhythmic music game intervention on the score of TGMD-3 coordination ability of ADHD children

**Figure 4:**
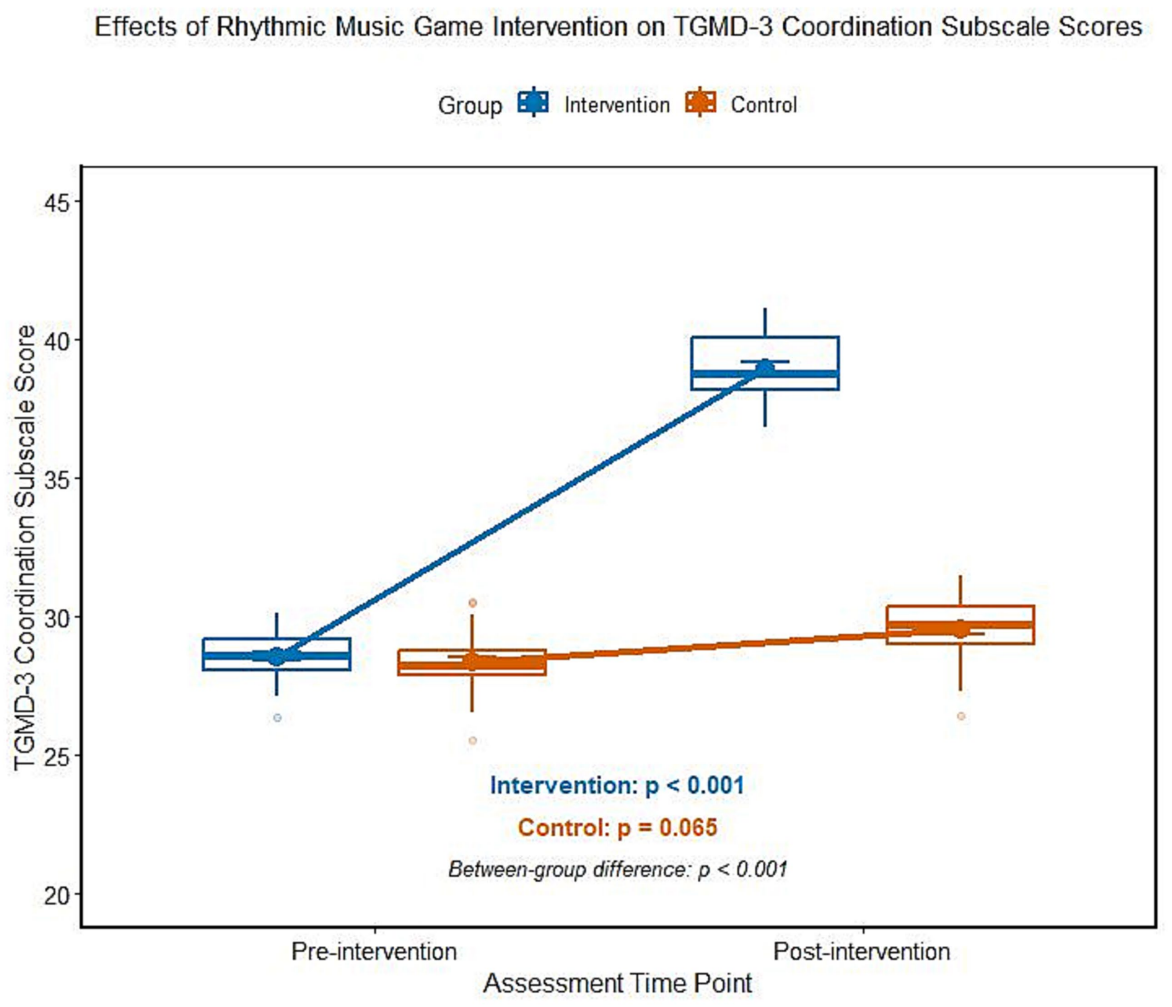
Comparison of TGMD-3 coordination ability score between two groups of ADHD children before and after intervention.

The analysis of variance of repeated measurements showed that there was a significant interaction between groups and time in the score of TGMD-3 coordination ability (*F* = 156.982, *p* < 0.001). After the intervention, the score of TGMD-3 coordination ability in the intervention group was significantly higher than that before the intervention, but there was no significant difference in the score of this dimension in the control group before and after the intervention, and the difference between the two groups was statistically significant (Table 5; Figure 4). According to the stratified analysis of medication history, there was no significant difference in the improvement of TGMD-3 coordination ability score between the non-medication subgroup and the drug withdrawal subgroup after intervention (*t* = 0.592, *p* = 0.556).

**Table 5:** Comparison of TGMD-3 coordination subscale scores in children with ADHD between the two groups before and after intervention (x̄ ± s, points).

Group	Before intervention	After intervention	Difference (after–before)
Intervention group (*n* = 32)	28.6 ± 4.2	38.9 ± 4.7	10.3 ± 3.5
Control group (*n* = 32)	28.3 ± 4.5	29.5 ± 4.3	1.2 ± 2.8
Between-group comparison (*t*-value)	0.301	9.026	13.826
Between-group comparison (*p*-value)	0.764	<0.001	<0.001

### 3.6 Influence of rhythmic music game intervention on ARS-5 score of ADHD children

The analysis of variance of repeated measurements showed that there was a significant interaction effect between groups and time on the total score of ARS-5 (*F* = 105.634, *p* < 0.001). After the intervention, the total score of ARS-5, the scores of attention deficit and hyperactivity impulse in the intervention group were significantly lower than those before the intervention (*p* < 0.001), but there was no significant difference in the scores of each dimension and the total score in the control group before and after the intervention (*p* > 0.05), and the difference between the two groups was statistically significant (*p* < 0.001). According to the stratified analysis of medication history, there was no significant difference in the reduction of the total score of ARS-5 between the non-medication subgroup and the drug withdrawal subgroup after intervention (*t* = 0.684, *p* = 0.497).

## 4 Discussion

### 4.1 Rhythmic music games improve the executive function of ADHD children

Executive dysfunction is the core cognitive feature of ADHD children, in which inhibition control and cognitive flexibility damage directly affect children’s attention regulation, behavioral constraints, and environmental adaptability (12). In this study, after the intervention of rhythmic music games, the number of semantic errors in SCWT was significantly reduced, and the completion time of TMT-B was significantly shortened, suggesting that this intervention can effectively improve children’s inhibition control and cognitive flexibility, which is consistent with the previous research conclusions of music intervention (13). From the perspective of neurocognitive mechanisms, rhythmic music games can effectively regulate the cooperative activities of the prefrontal lobe-striatum-cerebellum neural network through fixed beat stimulation and synchronous action training (14). From the perspective of neurocognitive mechanism, it is speculated that rhythmic music games may effectively regulate the cooperative activities of prefrontal cortex-striatum-cerebellum neural network through fixed beat stimulation and synchronous action training (15). As the core brain area of executive function, the abnormal functional connection between the prefrontal cortex and striatum is the key neural basis of ADHD children’s inhibition and control deficiency (16), and the task of rhythm perception and action synchronization may improve the information processing efficiency of the prefrontal cortex by enhancing the synchronization of neural oscillation in alpha and beta bands, and then enhance the inhibition and control ability (17). The improvement of TMT-B task also reflects the improvement of cognitive flexibility, which may be closely related to the beat change, multi-person cooperation and rhythm creation task in rhythm games. The cognitive strategy adjustment training brought by this kind of task may enhance the information integration ability between cerebral hemispheres and promote the efficiency of cognitive transformation (18). It is worth noting that after the control group only received routine health education, there was no significant change in all executive function indexes, which further confirmed the specific effect of rhythmic music game intervention, and its structured task design and multi-modal stimulation mode were obviously superior to the simple popularization of health knowledge (19). In this study, the subjects were divided into two subgroups according to the history of drug use: those who did not take drugs and those who stopped taking drugs. The results showed that there was no significant difference in the improvement degree of executive function indexes between the two subgroups after intervention, suggesting that the history of drug use did not significantly bias the intervention effect of this study, and the provisions on drug use in the inclusion criteria of this study did not lead to heterogeneous interference of the research population.

### 4.2 Effects of rhythmic music games on sensory motor ability of ADHD children

Abnormal sensory-motor integration is an easily overlooked functional defect of ADHD children, which is characterized by poor physical coordination and insufficient motor control ability, etc., and it interacts with executive function defects, aggravating children’s daily dysfunction (20). Compared with the previous rhythm-based training methods, the intervention scheme of this study is significantly innovative. It is not a simple rhythm synchronization training, but a three-dimensional task mode with step-by-step difficulty progression, which integrates rhythm synchronization, rhythm creation and multi-person cooperation. At the same time, combined with the visual feedback of tablet equipment and the precise linkage of limb movements, it strengthens the activation of the “auditory-motor” channel, and the phased design is more in line with the children’s cognitive and motor development laws, which is the key factor for the remarkable intervention effect of this study. The core mechanism of this result lies in the embodied cognitive characteristics of rhythmic music games. The potential mechanism of this result lies in the embodied cognitive characteristics of rhythmic music games. Rhythm, as the core element of music, may activate the pre-activation state of cerebral motor cortex through auditory channels, forming an “auditory-motor” linkage channel (21). ADHD children have neural signal delay in visual information processing and action preparation, and regular beat stimulation may be used as a “time anchor” for sensory and motor integration, reducing the delay of neural signal transmission and improving the accuracy and coordination of action execution (22). The intervention scheme of this study adopts a phased progressive design: the basic rhythm synchronous training in the adaptation period helps children to establish sensory motor mapping, the rhythm change and multi-person cooperative task in the promotion period strengthen the motor coordination ability, and the creation of complex rhythm in the consolidation period further promotes limb control and creative motor expression (23). This step-by-step training mode not only conforms to the development law of children’s motor skills but also maintains children’s participation enthusiasm through gamification design and ensures the sustainability of intervention effect (24). Compared with traditional sports intervention, rhythmic music games combine auditory stimulation, cognitive challenge, and social interaction and can activate multiple sensory motor channels at the same time. Its multi-modal integration advantage is more suitable for the functional improvement needs of ADHD children (25).

### 4.3 The practical value of rhythmic music game intervention

At present, the clinical intervention of ADHD is mainly drug therapy, but the side effects of stimulants and the risk of recurrence after withdrawal limit its long-term application, and the development and promotion of non-drug intervention has become a global consensus. The rhythmic music game intervention in this study has obvious clinical advantages: First, it is interesting and accessible. The intervention adopts tablet devices to carry customized game programs, which is in line with children’s gamification learning nature, and the intervention period is flexible and easy to operate, which can be carried out in various scenarios such as families and schools, effectively solving the problems of high threshold and low compliance of traditional intervention programs. Second, the multi-dimensional improvement effect is remarkable. This intervention is aimed at the two core defects of executive function and sensory motor ability of ADHD children, and can also effectively alleviate the core symptoms of ADHD, and realize the synergistic improvement of “cognition-movement-symptoms,” which has more comprehensive advantages than a single intervention (such as simple sports training or cognitive training). Third, the security is high. There is no drug participation in the whole intervention process, and training is only carried out through gamification tasks, without any adverse reactions. In this study, intervention compliance is clearly defined as course attendance rate, and the actual intervention compliance is ≥90%, indicating that the program is suitable for long-term application of ADHD children aged 6–12. From the perspective of public health, the low threshold of rhythmic music game intervention can reduce the difference of intervention accessibility of children from different regions and family backgrounds, which is in line with the core demand of “reducing inequality” in the sustainable development goal, especially suitable for ADHD intervention and promotion in areas with limited resources. This intervention program is jointly implemented by music therapists and pediatric rehabilitation therapists, and its multidisciplinary cooperation model provides a new practical paradigm for comprehensive intervention of ADHD, which can be used as an important part of a multidisciplinary intervention program and enrich the non-drug intervention system of ADHD.

There are also some design limitations in this study. In this study, the control group is only a blank control group receiving routine health education. Compared with the intervention group, the total intervention duration (the total intervention duration of the control group is 80 min/8 weeks, and the total intervention duration of the intervention group is 960 min/8 weeks), the professional interaction form (the control group has no face-to-face interaction online). There are significant differences in the frequency of peer contact (no peer contact in the control group, peer cooperation in the intervention group). These nonspecific factors (structured activity arrangement, face-to-face professional guidance, positive peer social interaction, etc.) may work together with the core elements of rhythm music, which will have an impact on the research results. It cannot be completely ruled out that they can improve the executive function, sensory motor ability and core symptoms of ADHD children alone. In view of this limitation, it is necessary to set up an active control group in future research (using non-rhythmic traditional video games for equal duration and equal forms of intervention), balance the non-specific variables such as intervention dose, professional interaction and peer contact between the two groups, and more accurately separate and verify the core intervention effect of rhythmic music games.

### 4.4 Research limitations and future direction

Although this study has achieved positive results, there are still some limitations: first, there is a serious imbalance in the intervention dose between the intervention group and the control group. The intervention group received about 960 min of structured and therapist-directed interactive training, while the control group only received 80 min of passive health education. The intervention form, duration and interaction between the two groups are significantly different, so it is impossible to attribute the observed intervention effect to the core components of rhythmic music at present, which is the main limitation of this study; Secondly, the follow-up period of this study is short, and the long-term sustainability of the intervention effect is not discussed. In the future, a longer follow-up study is needed to verify the long-term effect of the intervention of rhythmic music games. Thirdly, this study adopts the convenient sampling method, and the subjects are only from the pediatric psychological outpatient department of our hospital. The sample source is single. Although the baseline is moderate and severe ADHD symptoms, there may still be selection bias and sample representativeness is limited. The follow-up study should expand the sample size and adopt a multi-center and random sampling design, and at the same time report the recruitment source of the subjects, the severity of baseline symptoms and other information in detail to enhance the extrapolation of the research results. Fourthly, this study did not deeply discuss the heterogeneous response of different ADHD subtypes (attention deficit type, hyperactivity type and mixed type) to the intervention effect. In the future, personalized intervention programs can be designed for children of different subtypes to achieve accurate intervention. Fifth, although this study confirmed the comprehensive intervention effect of rhythmic music games, it is impossible to completely separate the role of a single factor because of the integration of music, sports, cognition and other factors. The follow-up study can set up different experimental groups by splitting the intervention factors to clarify the independent role and synergistic effect of each factor; Sixth, the mechanism explanation of the results from the perspective of neural oscillation and brain network in this study is based on the theoretical assumptions of the existing literature, and no neurophysiological data such as fMRI and EEG are collected to verify it, and the related mechanism inference is lack of direct experimental evidence.

Based on the above limitations, this study puts forward the following specific directions for future research: (1) Follow-up research needs to set up an active control group (such as non-rhythmic game intervention) that matches the intervention dose and interaction form of the intervention group, balance the intervention conditions between groups, clarify the specific role of rhythmic music components in the intervention effect, and verify the causal relationship of the intervention more strictly; (2) Conduct multi-center and large-sample randomized controlled trials, adopt scientific sampling methods, and report the recruitment source, baseline symptom severity, demographic characteristics and other information in detail to enhance the external validity of the research results; (3) Design personalized intervention programs for different ADHD subtypes, analyze the heterogeneous influence of subtypes on intervention effect, and realize the accuracy of intervention; (4) Combining with neuroimaging techniques such as fMRI and EEG, we will carry out mechanism research, and transform the related explanations such as neural oscillation and brain network from theoretical assumptions to conclusions supported by experimental data, reduce speculative mechanistic claims, and make the mechanism explanation strictly conform to the behavioral results; (5) Extend the follow-up period, explore the long-term sustainability of the intervention effect of rhythmic music games, and provide evidence for its long-term clinical application.

## 5 Conclusion

To sum up, under the intervention design of this study, the rhythmic music game intervention shows a positive role in improving the executive function and sensory motor ability of ADHD children and alleviating their core symptoms, and has multiple advantages such as fun, accessibility and safety. Its structured and stepped three-dimensional task design is remarkably innovative, and the multi-modal integration characteristics meet the functional improvement needs of ADHD children. However, due to the core limitation of the imbalance of intervention dose between the intervention group and the control group in this study, it is not clear whether the effect is specifically attributed to rhythmic music components, and the causal relationship of related intervention effects needs further verification in subsequent studies. In this study, the sample size was determined by sample size calculation, and the selection bias was reduced by block randomization and distribution concealment. The positive effect of intervention was verified by repeated measurement analysis of variance, and the medication history did not have obvious bias on the intervention effect. This intervention program provides a new practical path for non-drug intervention of ADHD children, and also provides a practical reference for the development of multidisciplinary collaborative intervention model, which improves children’s health level and has important practical value and promotion potential. In the follow-up, it is necessary to further verify its core effect and mechanism by optimizing research design and supplementing mechanism data.

## Data Availability

The original contributions presented in the study are included in the article/supplementary material, further inquiries can be directed to the corresponding author.
